# Effect of Ningxiang pig-derived *Lactobacillus reuteri* supplementation on the growth performance, lipid metabolism, and gut microbiota of finishing pigs

**DOI:** 10.1186/s40813-025-00474-1

**Published:** 2025-11-25

**Authors:** Qian Xie, Mei Yang, Qing Duanmu, Luya Feng, Luling Liu, Yulong Yin, Bi E Tan, Jiashun Chen

**Affiliations:** 1 Key Laboratory of Hunan Province for the Products Quality Regulation of Livestock and Poultry, College of Animal Science and Technology, Hunan Agricultural University, Changsha, Hunan 410128 P. R. China; 2 Yuelushan Laboratory, Changsha, Hunan 410128 P. R. China; 3 Key Laboratory of Agro-Ecological Processes in Subtropical Region, National Engineering Laboratory for Pollution Control and Waste Utilization in Livestock and Poultry Production, Institute of Subtropical Agriculture, Chinese Academy of Sciences, Changsha, Hunan 410125 P. R. China; 4 Huizhou Engineering Vocational College, Huizhou, Guangdong 516023 P. R. China

**Keywords:** Lactobacillus reuteri, Growth performance, Lipid metabolism, Gut microbiota

## Abstract

**Background:**

While the pivotal role of the gut microbiota in lipid metabolism has been well established, the specific contributions of bacterial strains isolated from native Chinese pig breeds to lipid metabolic regulation remain largely uninvestigated. The purpose of this research was to investigate the effects of Ningxiang pig-derived *L. reuteri XY227* supplementation on growth performance, lipid metabolism, and gut microbiota in finishing pigs.

**Methods:**

Sixteen healthy Duroc × Landrace × Yorkshire (DLY) finishing pigs, with an initial body weight of 63.36 ± 1.28 kg, were randomly assigned to two groups, each with eight replicates. The dietary treatments included a basal diet and a basal diet supplemented with 0.4% *L. reuteri XY227* (1 × 10¹¹ CFU/kg of basal diet). The experiment lasted for 50 days.

**Results:**

Supplementation with *L. reuteri XY227* significantly increased the final body weight (FBW) and average daily gain (ADG) (*P* < 0.05). Dietary supplementation with *L. reuteri XY227* significantly reduced the concentrations of C12:0, C18:1n9c, C20:2, and total monounsaturated fatty acids (MUFA) (*P* < 0.05) in the dorsal subcutaneous adipose tissue. In the *L. reuteri XY227* group, mRNA expressions of sterol regulatory element binding protein-1c (*SREBP-1 C)*, peroxisome proliferator-activated receptor γ (*PPARγ*), and acetyl CoA carboxylase (*ACC*) in subcutaneous adipose tissue were notably reduced (*P* < 0.05). The expression of fatty acid translocase (*FAT/CD36*) was significantly elevated (*P* < 0.05). *L. reuteri XY227* supplementation increased Cluster of differentiation 36 (*CD36*) mRNA expression in the jejunum and improved jejunal and ileal the height of villi (*P* < 0.05). Diet supplemented with *L. reuteri XY227* significantly boosted the abundance of *Lactobacillus*,* Enterococcus*,* and Limosilactobacillus* in the ileum mucosa and the valerate content in the colon (*P* < 0.05).

**Conclusions:**

Collectively, these findings indicate that *L. reuteri XY227* supplementation improves growth performance and modulates lipid metabolism, possibly achieved through improvements in intestinal morphology and alteration of the gut microbial community.

## Introduction

Over the past decade, the critical role of gut microbiota in lipid metabolism has been highlighted. Dietary-induced variations in host metabolism are partially attributed to gut microbiota, which has been demonstrated in germ-free mice [1]. Metabolites produced by gut microbiota, such as trimethylamine, secondary bile acids, and short-chain fatty acids (SCFAs), can mediate this effect on host lipid metabolism [2]. The identification of bacterial strains directly involved in lipid metabolism is scarce [3, 4]. Recent evidence suggests that *Prevotella copri*, isolated from the feces of Duroc pigs with extremely low lean meat percentage, may promote fat accumulation in the host [5]. However, the functional roles of many bacterial strains isolated from native Chinese pig breeds remain largely unexplored.

*Lactobacillus reuteri* (*L. reuteri*) can impact lipid metabolism and produce bioactive compounds, such as lactate, reutericyclin, and bile salt hydrolases, which are crucial for maintaining host physiology [6, 7]. Evidence from the literature suggests that *L. reuteri* treatment improves lipid metabolism, thereby preventing metabolic disorders and intestinal injury in mice on a high-fat diet [8]. It has been reported that daily administration of *L. reuteri* restructured gut microbial composition and enhanced the production of beneficial metabolites [9]. In our previous study, supplementation of *L. reuteri* XY227 demonstrated potential probiotic attributes in both in vitro and in rat models [10]. In our study, we aimed to evaluate the in vivo effectiveness of this newly identified probiotic strain under practical pig production conditions, as research on its application in finishing pigs remains limited. Therefore, the aim of this study was to assess the effects of *L. reuteri XY227* supplementation on growth performance, lipid metabolism, and gut microbiota in finishing pigs, providing a theoretical foundation for the further application and development of *L. reuteri XY227* in the pig industry.

## Materials and methods

### Experimental design

All procedures adhered to the Animal Ethics Committee of Hunan Agricultural University, China (CACAHU 2023 − 0317) guidelines. Sixteen healthy Duroc×Landrace×Yorkshire (DLY) finishing pigs, each initially weighing 63.36 ± 1.28 kg, were randomly assigned to two groups (*n* = 8 per group, 1 pig per replicate): the control group (NC, basal diet) and the *L. reuteri* XY227-supplemented group (LR, basal diet supplemented with 0.4% *L. reuteri* XY227). All pigs were housed under identical environmental conditions in individual cages, with ad libitum access to food and water throughout the study period. The remaining feed was weighed, and the feed intake was recorded after each feeding. The initial and final body weights (FBW) of each pig were measured. Average daily gain (ADG), average daily feed intake (ADFI), and feed-to-gain ratio (F: G) were calculated. The experiment lasted for a duration of 50 days. The basal diet met the NRC (2012) requirements for finishing pigs, as detailed in Table 1. Net energy (NE) was calculated according to the Feed Composition and Nutritive Values in China (2020). The crude protein (CP) contents in diet were measured using kjeldahl analysis system (Gerhardt VAP450, Germany) according to the method in GB/T 6432 − 2018. The total calcium (method 984.01), and total phosphorus (method 965.17), crude fat (method 920.39), and ashing (method 942.05) in diet were measured according to the procedures of the AOAC (2006). Neutral detergent fiber (NDF) and acid detergent fiber (ADF) in the feed were determined using the ANKOM 200i Fiber Analyzer (ANKOM Technology, America) following the standard methods in the determination of NDF in Feeds (GB/T 20806 − 2022) and the determination of ADF in Feeds (NY/T 1459–2022), respectively.

**Table 1:** Ingredients and nutrient levels of experimental basal diet (%, as-fed basis)

Ingredient	Content
Corn	68.00
Soybean meal	18.00
Wheat bran	8.00
Soybean oil	2.00
Wheat middling	1.30
Limestone	0.90
Dicalcium phosphate	0.80
Premix^1^	1.00
Total	100.00
Nurient levels^2^	
DE, (MJ/kg)	13.86
Crude protein	14.68
Crude fat	4.55
Ash	4.61
NDF	13.28
ADF	4.16
Calcium	0.62
Total phosphorus	0.49
Available phosphorus	0.29

^1^Supplied per kilogram of diet: vitamin A, 6500 IU; vitamin D3, 2000 IU; vitamin E, 16 IU; vitamin K3, 2 mg; vitamin B1, 2 mg; vitamin B2, 5 mg; vitamin B6, 1.6 mg; vitamin B12, 0.015 mg; biotin, 0.12 mg; pantothenic acid, 10 mg; folic acid, 1 mg; nicotinamide, 20 mg; Cu, 15 mg; Fe, 120 mg; Mn, 40 mg; Zn, 70 mg; I, 0.8 mg; Se, 0.4 mg; Co, 0.25 mg; Cr, 0.2 mg

^2^ Nutrient level was calculated value

DE: digestible energy; NDF: neutral detergent fiber; ADF: acid detergent fiber

### Preparation of *L. reuteri XY227*

Fecal samples from Ningxiang pigs were collected, stored at 4 °C, and promptly transported to the laboratory for bacterial isolation. One-gram fecal samples were suspended in normal saline solution and serially diluted to 10^− 6^~10^− 8^. The dilutions were spread on MRS plates, and single colonies were repeatedly subcultured at least thrice to obtain pure cultures and transferred to the MRS broth to identify the strain. Isolated *L. reuteri XY227* was stored at the China Center for Type Culture Collection (CCTCC M 20231546 XY227). The strain was processed into powder by CRVAB Bio-technology Co., Ltd. *L. reuteri XY227* were lyophilized with a viable count of 2.5 × 10^10^ CFU/g in power for subsequent feeding experiments.

### Sample collection and analysis

At the end of the experiment, 5 mL of blood was collected from the anterior vena cava of pigs in a vacuum blood collection tube, and after standing for 30 min, the serum was centrifuged at 3500 r/min for 10 min, and the serum was divided into 1.5 mL sterile centrifuge tubes and stored at -80 °C for testing. Twelve hours before sampling, pigs were fasted. On the morning of day 50, they were euthanized by exsanguination following electrical stunning. The dorsal subcutaneous adipose tissue, jejunum, and ileum were immediately excised and flash-frozen in liquid nitrogen for further analysis. Additionally, sections of subcutaneous adipose tissue, 2-cm section from the middle of the jejunum and ileum were fixed with either fat fixative solution (Servicebio, Cat. No. G1119) or 4% paraformaldehyde, as appropriate, for histological staining analysis. A portion of the ileal segment was rinsed with saline, the surface mucosal layer was scraped with a slide, and the samples were stored at -80 °C to analyze the gut microbiota.

### Serum biochemical parameters

The concentrations of glucose (GLU); alkaline phosphatase (ALP); alanine aminotransferase (ALT); aspartate aminotransferase (AST); total protein (TP); blood urea nitrogen (BUN); albumin (ALB); globulin (GLB) in serum were measured using an automatic biochemical analyzer (KHB 450, Shanghai Kehua Bio-Engineering Co., Ltd., Shanghai, China).

### Fatty acid composition

Fatty acid profiles in dorsal subcutaneous adipose tissue were analyzed following a previously described method [11]. Lipids from freeze-dried dorsal subcutaneous adipose samples were extracted using a benzene-petroleum ether (1:1) mixed solvent. The extracted lipids were saponified with a 0.4 mol/L sodium hydroxide methanol solution to obtain lipid methyl esters. Fatty acid analysis was performed using a gas chromatography system (Agilent 8890, Agilent Technologies, Santa Clara, CA, USA).

### RNA extraction and quantification

Total RNA was extracted from tissues using RNAiso Plus (Cat # 9109, Takara Biomedical Technology, Beijing, China). RNA concentration and purity were measured with a NanoDrop One (Thermo Fisher, Massachusetts, USA). cDNA was synthesized using the Evo M-MLV RT Kit with gDNA Clean for qPCR (AG11705, Accurate Biotechnology, Hunan, China) following the manufacturer’s instructions. Real-time PCR was conducted using the SYBR Green Premix Pro Taq HS QPCR Kit (AG11701, Accurate Biotechnology, Hunan, China) on a LightCycler480II system (Roche Diagnostics GmbH, Mannheim, Germany). The relative mRNA expression levels were calculated using the 2^−ΔΔCT^ method as described previously [12]. A list of primer sequences is provided in Table 2.

**Table 2:** Primers used for quantitative real-time PCR

Genes	Primers	Sequences (5′ to 3′)
*LPL*	Forward	CTCGTGCTCAGATGCCCTAC
	Reverse	GGCAGGGTGAAAGGGATGTT
*HSL*	Forward	CACAAGGGCTGCTTCTACGG
	Reverse	AAGCGGCCACTGGTGAAGAG
*CPT1*	Forward	GACAAGTCCTTCACCCTCATCGC
	Reverse	GGGTTTGGTTTGCCCAGACAG
*SREBP1-C*	Forward	GCGACGGTGCCTCTGGTAGT
	Reverse	CGCAAGACGGCGGATTTA
*PPARγ*	Forward	CAGGCCACCACCGCAGATT
	Reverse	CAACCATGGTCACCTCGCTAAA
*ACLY*	Forward	GGCCTTTCGTAGAGAGCAGG
	Reverse	CCATCCAGGGTGAGGTTGAC
*ACC*	Forward	CGGAATATCCAGAAGGCCGA
	Reverse	CCAGTCCGATTCTTGCTCCA
*C/EBP-α*	Forward	GCAGAGATCCCTATAAACCAGC
	Reverse	TTCAAAGCCCCAAGTCCC
*CD36*	Forward	CTGGTGCTGTCATTGGAGCAGT
	Reverse	CTGTCTGTAAACTTCCGTGCCTGTT
*FABP4*	Forward	CAGGAAAGTCAAGAGCACCA
	Reverse	TCGGGACAATACATCCAACA
*FATP1*	Forward	ACCACTCCTACCGCATGCAG
	Reverse	CCACGATGTTCCCTGCCGAGT
*GLUT4*	Forward	CGAGGCAGGACGTTTGACC
	Reverse	CTCCAGTTCTGTGCTGGGTTTC
*β-actin*	Forward	CTACGCCAACACGGTGCTGTC
	Reverse	CTCCTGCTTGCTGATCCACATCTG

*LPL*: lipoprotein lipase; *HSL*: hormone-sensitive lipase; *CPT-1*: carnitine palmitoyltransferase 1; *SREBP1-C*: sterol regulatory element binding protein-1c; *PPARγ*: peroxisome proliferator-activated receptor γ; *ACLY*: ATP-citrate lyase; *ACC*: acetyl CoA carboxylase; *C/EBP-α*: CCAAT enhancer binding protein α; *CD36*: Cluster of differentiation 36; *FABP4*: fatty acid binding protein 4; *FATP1*: fatty acid transport protein 1; *GLUT4*: glucose transporter type 4

### Histological analysis

Morphology of the jejunum, ileum, and dorsal subcutaneous adipose tissue was analyzed by embedding samples in paraffin, sectioning jejunum and ileum into 4 μm long and dorsal subcutaneous adipose tissue into 4 μm thick using a microtome (Leica Biosystems Nussloch GmbH, Nussloch, Germany) and staining with hematoxylin and eosin (H&E). The sections were examined using an inverted fluorescence microscope (Axio Vert A1, Carl Zeiss AG, Jena, Germany). Crypt depth, villus height, and adipocyte number and diameter were quantified by automatic digital image analysis using the ImageJ software (National Institutes of Health, Bethesda, MD, USA).

### Analysis of the gut microbiota

Total microbial genomic DNA was extracted from the ileum mucosa using the CTAB method following the manufacturer’s protocol. The V3–V4 regions of the bacterial 16 S rDNA genes were amplified with universal primers 341 F (5’-CCTACGGGNGGCWGCAG-3’) and 805R (5’-GACTACHVGGGTATCTAATCC-3’). Sequencing was performed on the Illumina NovaSeq platform following the manufacturer’s protocol. Dereplication with DADA2 provided feature tables and sequences, which were normalized for feature abundance using the relative abundance of each sample in the SILVA database (release 138). Alpha and beta diversity indices were calculated using QIIME 2. The Shannon index and Simpson index represented alpha diversity. Principal coordinate analysis (PCoA) was performed with weighted UniFrac distance metrics. Differential taxa were identified using linear discriminant analysis effect size (LEfSe). Sequence data are available in the SRA database (PRJNA1063880).

### Quantification of short-chain fatty acid profiles

Colonic contents were dissolved and homogenized in ultrapure water. After centrifugation at 15,000 g for 15 min at 4 °C, the supernatant was collected and diluted with 25% metaphosphoric acid (9:1). The mixture was filtered through a 0.22-µm sterile membrane, placed in a 2 mL screw-cap vial, and subjected to gas chromatography analysis (Agilent 8890, Agilent Technologies, Santa Clara, CA, USA).

### Statistical analysis

The experimental unit was an individual pig (*n* = 8 per group), as each pig was housed in an independent cage and treated as a single replicate. Normally distributed of the data was tested using the Shapiro–Wilk test, and Levene’s test was utilized to assess the homogeneity of variances. Data was analyzed by Student’s t-test using SPASS 25.0, and the results are presented as the mean and standard error of the mean (SEM). Graphs were plotted using GraphPad Prism 9.5. Differences were considered statistically significant at *P* < 0.05.

## Results

### Growth performance

Detailed data on the growth performance of finishing pigs are presented in Table 3. Pigs in the LR group showed significantly higher FBW and ADG compared to the NC group (*P* < 0.05). Additionally, *L. reuteri* XY227 supplementation tended to increase ADFI (*P* = 0.088). However, no significant difference was observed in the feed-to-gain (F: G) ratio between the two groups.

**Table 3:** Effect of dietary *L. reuteri XY227* supplementation on growth performance of the finishing pigs (*n* = 8)

Item	NC	LR	SEM	*P*-value
IBW, kg	63.29	63.43	0.710	0.845
FBW, kg	113.24^b^	120.55^a^	2.782	0.022
ADG, kg/d	1.00^b^	1.14^a^	0.049	0.013
ADFI, kg/d	3.08	3.35	0.145	0.088
F: G ratio	3.08	2.94	0.082	0.116

NC: basal diet; LR: basal diet supplemented with 0.4% *L. reuteri XY227* (1 × 10^11^ CFU/kg of basal diet); IBW: initial body weight; FBW: final body weight; ADG: average daily gain; ADFI: average daily feed intake

^a, b^ Values within a row with different superscripts differ significantly at *P* < 0.05

### Concentration of serum biochemical indicators

Levels of TP, ALP, ALT, AST, ALB, GLB, BUN, and GLU were measured to assess the impact of *L. reuteri XY227* supplementation on serum biochemistry. As shown in Table 4, the administration of *L. reuteri XY227* tended to increase GLB concentration compared with the NC group (*P =* 0.072). However, there were no significant effects on serum levels of TP, ALP, ALT, AST, ALB, BUN, and GLU.

**Table 4:** Effects of *L. reuteri XY227* on the serum biochemical parameters of finishing pigs (*n* = 8)

Item	NC	LR	SEM	*P*-value
GLU, mmol/L	7.04	8.80	1.444	0.240
ALP, U/L	133.13	149.49	12.914	0.222
ALT, U/L	55.09	62.92	5.271	0.158
AST, U/L	40.38	46.23	6.684	0.394
AST/ALT	0.72	0.72	0.096	0.931
TP, g/L	67.50	75.30	5.810	0.196
BUN, mmol/L	5.64	5.68	0.623	0.943
ALB, g/L	46.34	52.61	4.741	0.203
GLB, g/L	20.11	22.80	1.403	0.072
ALB/GLB	2.22	2.31	0.194	0.640

NC: basal diet; LR: basal diet supplemented with 0.4% *L. reuteri XY227* (1 × 10^11^ CFU/kg of basal diet); GLU: glucose; ALP: alkaline phosphatase; ALT: alanine aminotransferase; AST: aspartate aminotransferase; TP: total protein; BUN: blood urea nitrogen; ALB: albumin; GLB: globulin

^a, b^ Values within a row with different superscripts differ significantly at *P* < 0.05

### Fatty acid composition of the dorsal subcutaneous adipose tissue

Fatty acid profiles of the dorsal subcutaneous adipose tissue were analyzed. Oleic acid (C18:1n9c), palmitic acid (C16:0), and linoleic acid (C18:2n6c) were the most abundant fatty acids (Table 5). Dietary supplementation with *L. reuteri XY227* significantly reduced the concentrations of C12:0, C18:1n9c, C20:2, and MUFA (*P* < 0.05). Moreover, a decreasing trend was observed for C8:0, C14:0, C18:2n6c, C18:3n3, and total polyunsaturated fatty acids (PUFA).C12:0 (0.05 < *P* < 0.10).

**Table 5:** Effects of *L. reuteri XY227* on the fatty acid profile of dorsal subcutaneous adipose tissue in finishing pigs (g/100 g dorsal subcutaneous adipose tissue, *n* = 8)

Item	NC	LR	SEM	*P*-value
C8:0	0.020	0.017	0.002	0.064
C10:0	0.056	0.051	0.003	0.117
C12:0	0.065^a^	0.057^b^	0.003	0.029
C14:0	1.059	0.951	0.052	0.07
C15:0	0.027	0.027	0.004	0.987
C16:0	21.377	19.571	1.108	0.141
C16:1	1.165	1.018	0.111	0.208
C17:0	0.177	0.181	0.032	0.915
C17:1	0.123	0.127	0.022	0.842
C18:0	12.576	11.723	0.875	0.346
C18:1n9c	32.887^a^	29.760^b^	1.447	0.049
C18:2n6t	0.025	0.024	0.002	0.782
C18:2n6c	14.554	12.778	0.853	0.056
C20:0	0.243	0.237	0.017	0.708
C18:3n6	0.069	0.064	0.005	0.263
C20:1	0.866	0.827	0.041	0.36
C18:3n3	0.749	0.664	0.040	0.051
C21:0	0.020	0.031	0.005	0.073
C20:2	0.784^a^	0.680^b^	0.047	0.043
C22:0	0.029	0.023	0.004	0.163
C20:3n6	0.080	0.076	0.008	0.583
C22:1n9	0.023	0.022	0.001	0.112
C20:3n3	0.125	0.110	0.007	0.053
C20:4n6	0.180	0.169	0.017	0.549
SFA	35.647	32.860	1.961	0.189
MUFA	35.064^a^	31.754^b^	1.539	0.049
PUFA	16.566	14.566	0.966	0.057

NC: basal diet, LR: basal diet supplemented with 0.4% *L. reuteri XY227* (1 × 10^11^ CFU/kg of basal diet). SFA: saturated fatty acids; MUFA: monounsaturated fatty acid; PUFA: polyunsaturated fatty acid

^a, b^ Values within a row with different superscripts differ significantly at *P* < 0.05

### Lipid metabolism in the dorsal subcutaneous adipose tissue

**Fig. 1:**
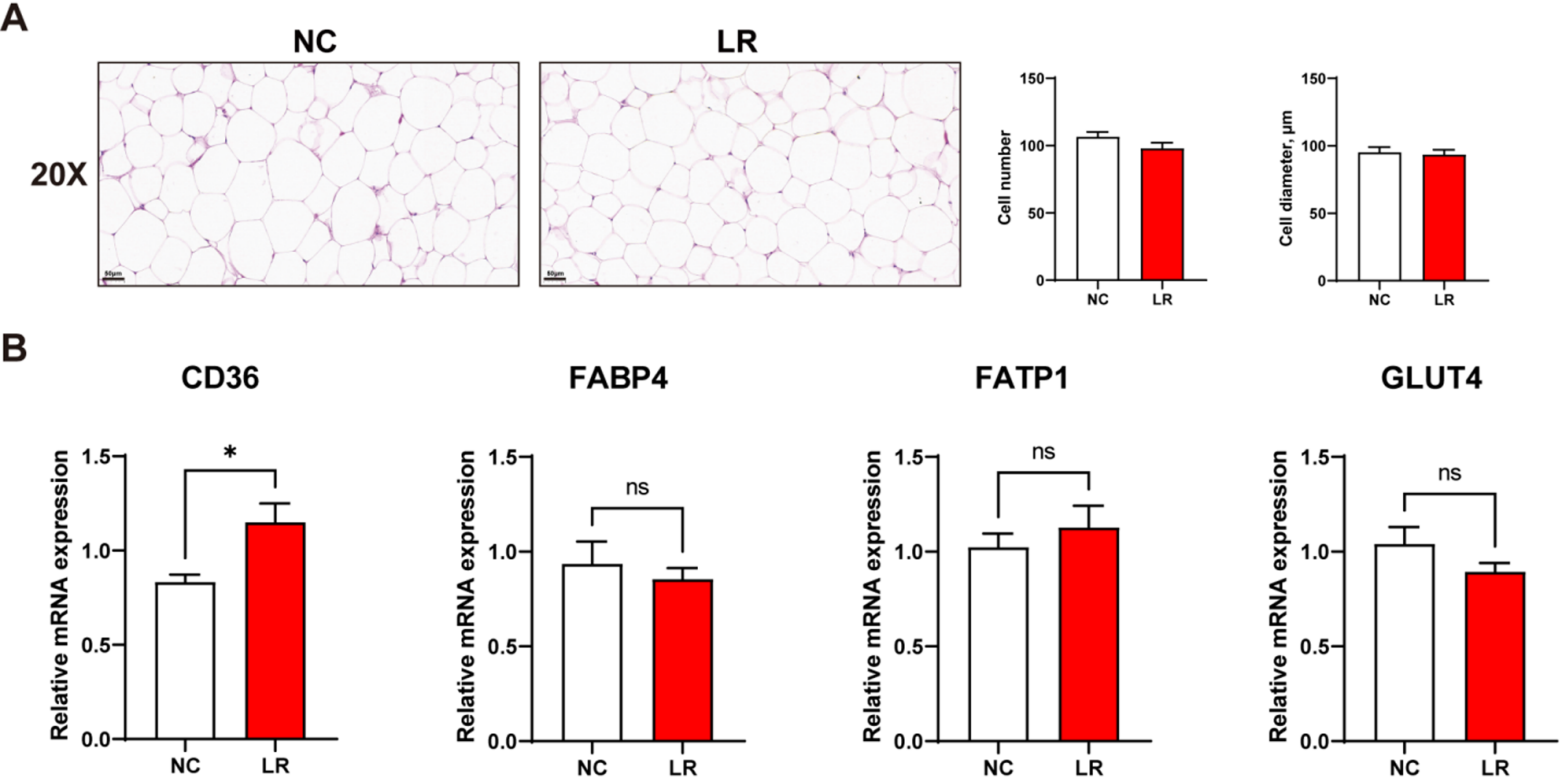
The expression of fatty acid metabolism in dorsal subcutaneous adipose tissue of finishing pigs (*n* = 8). (**A**) Representative images of dorsal subcutaneous adipose tissue H&E staining, and its histopathological scores of dorsal subcutaneous adipose tissue (staining magnification: 20×; scale bar: 50 μm). (**B**)The mRNA expression of fatty acid transport genes. NC: basal diet, LR: basal diet supplemented with 0.4% *L. reuteri XY227* (1 × 10^11^ CFU/kg of basal diet); *CD36*: Cluster of differentiation 36; *FABP4*: fatty acid binding protein 4; *FATP1*: fatty acid transport protein 1; *GLUT4*: glucose transporter type 4. Data are presented as means ± SEM. *P < 0.05, **P < 0.01, ***P < 0.001

There were no significant differences in the number and diameter of adipocytes between the *L. reuteri XY227* treatment group and the NC group (Fig. 1A, *P* > 0.05). We examined the expression of genes associated with fatty acid transport in subcutaneous adipose tissue, including *CD36*, fatty acid binding protein 4 (*FABP4*), fatty acid transport protein 1 (*FATP1*), and glucose transporter type 4 (*GLUT4*), to understand how *L. reuteri XY227* improved the fatty acid profile. *L. reuteri XY227* supplementation upregulated *CD36* mRNA expression compared with that in the NC group (Fig. 1B, *P* < 0.05). However, there were no significant differences in the mRNA expressions of *FABP4*, *FATP1*, and *GLUT4* between the LR and NC groups (Fig. 1B, *P* > 0.05).

**Fig. 2:**
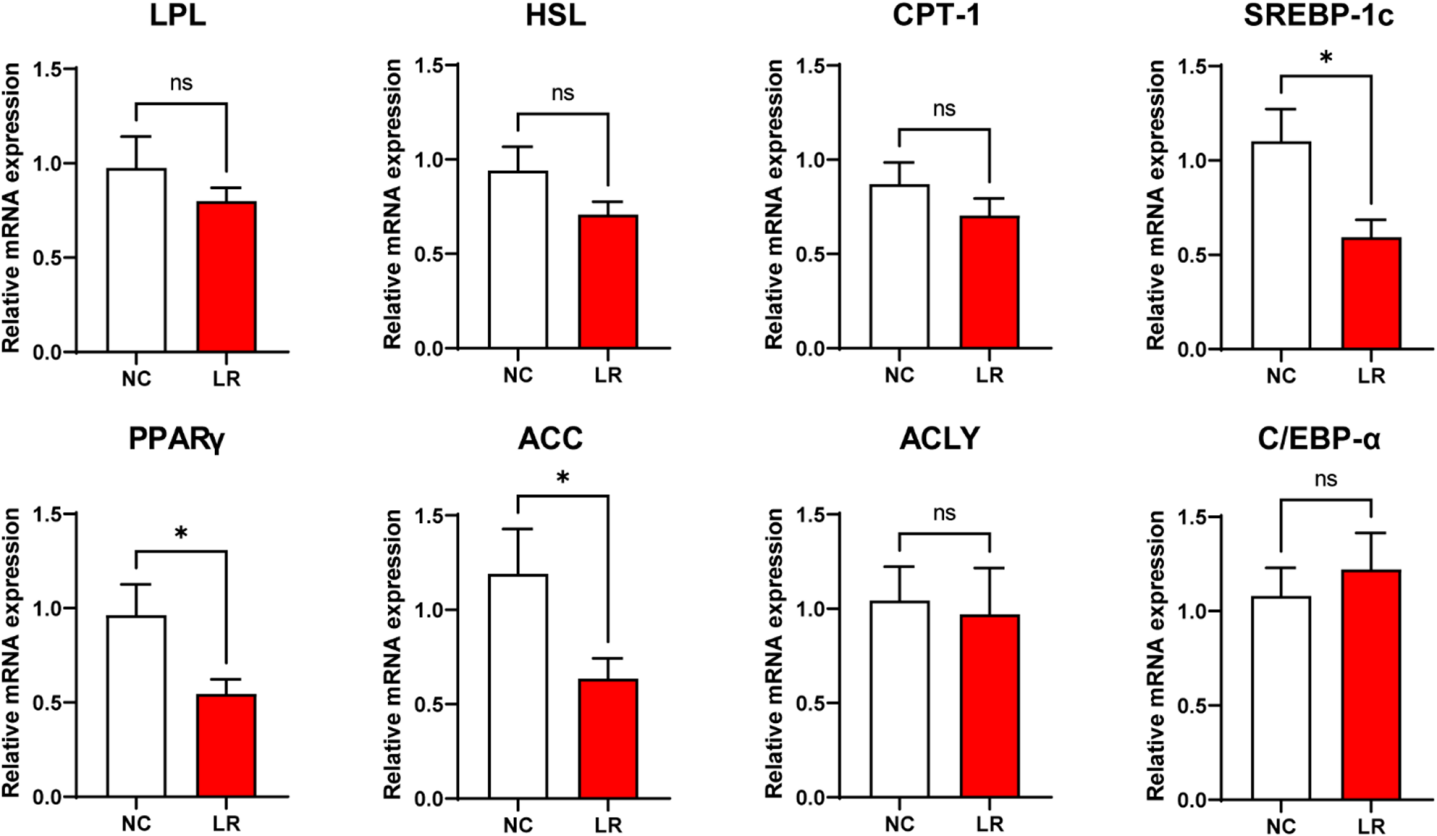
The mRNA expression of lipid metabolism in dorsal subcutaneous adipose tissue of finishing pigs (*n* = 8). NC: basal diet, LR: basal diet supplemented with 0.4% *L. reuteri XY227* (1 × 10^11^ CFU/kg of basal diet). Data are presented as means ± SEM. * *P* < 0.05, ** *P* < 0.01, *** *P* < 0.001. *LPL*: lipoprotein lipase; *HSL*: hormone-sensitive lipase; *CPT-1*: carnitine palmitoyltransferase 1; *SREBP1-C*: sterol regulatory element binding protein-1c; *PPARγ*: peroxisome proliferator-activated receptor γ; *ACLY*: ATP-citrate lyase; *ACC*: acetyl CoA carboxylase; *C/EBP-α*: CCAAT enhancer binding protein α

We analyzed the expression of several genes related to lipid metabolism (Fig. 2) to elucidate the molecular mechanisms underlying *L. reuteri XY227* action in lipid metabolism. *L. reuteri XY227* administration reduced the relative mRNA expression of adipocyte synthesis-related genes, including *SREBP1-C*, *PPARγ*, *ACC*, compared with the control group (*P* < 0.05).

### Fatty acid uptake and transport in the intestine

**Fig. 3:**
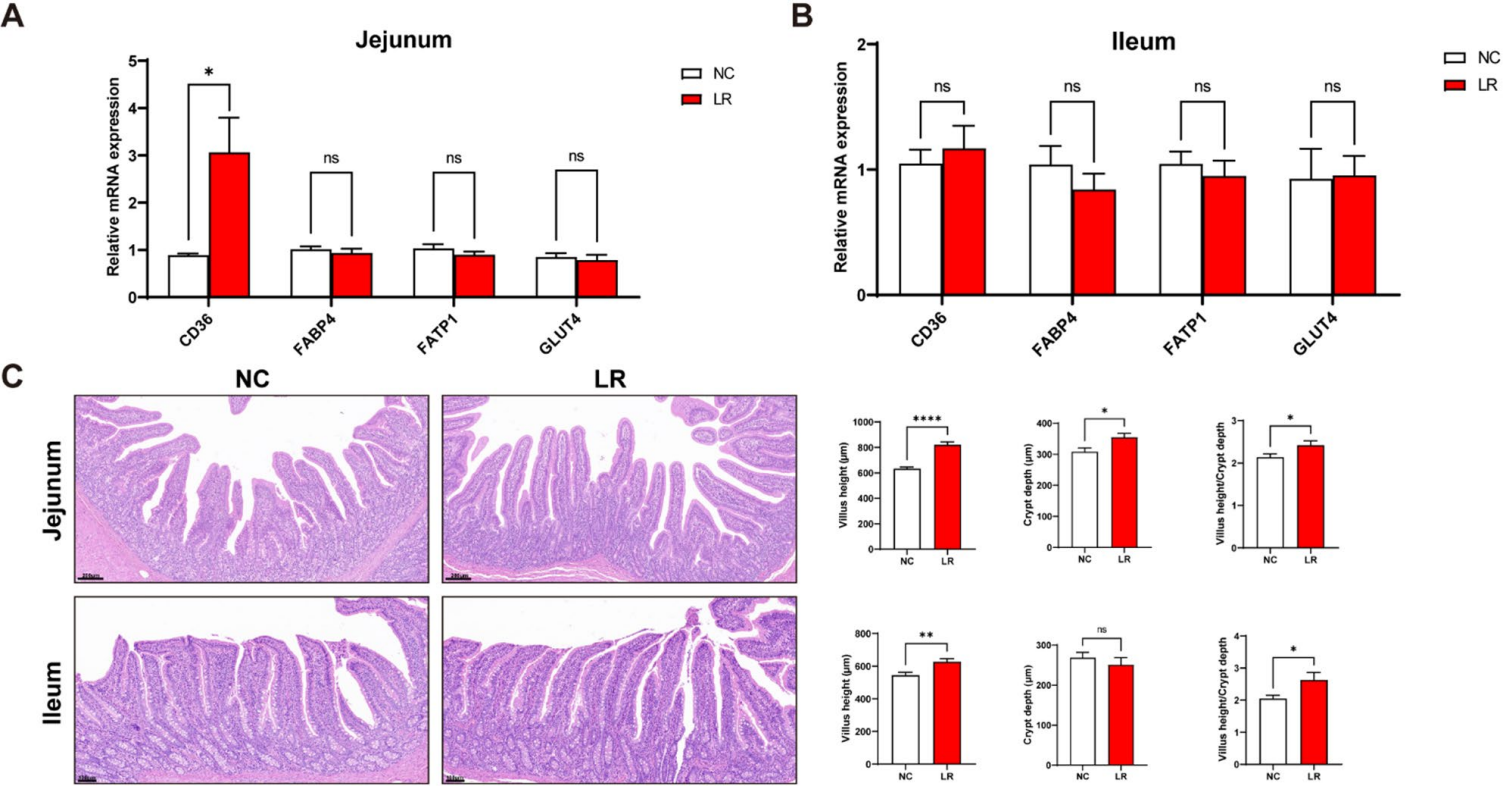
The differential expression levels of those genes involved in lipid transport in the intestinal (*n* = 8). (**A**) The mRNA expression of fatty acid transport genes in jejunum. (**B**) The mRNA expression of fatty acid transport genes in ileum. (**C**) Images of representative H&E-stained jejunal (staining magnification: 7×; scale bar: 200 μm) and ileal (staining magnification: 10×; scale bar: 100 μm) sections and its villus height and crypt depth. NC: basal diet, LR: basal diet supplemented with 0.4% *L. reuteri XY227* (1 × 10^11^ CFU/kg of basal diet); *CD36*: Cluster of differentiation 36; *FABP4*: fatty acid binding protein 4; *FATP1*: fatty acid transport protein 1; *GLUT4*: glucose transporter type 4. Data are presented as means ± SEM. *P < 0.05, **P < 0.01, ***P < 0.001

As shown in Fig. 3A, the mRNA expression of *CD36* in the ileum was significantly higher in the LR group compared with the NC group (*P* < 0.05). However, there were no differences in the mRNA levels of *FABP4*, *FATP1*, and *GLUT4* in the jejunum and ileum between the two groups (Fig. 3A-B). Figure 3C illustrates a significant increase in the height of both jejunal (*P* < 0.001) and ileal villi (*P* < 0.01) in the LR group. *L. reuteri XY227* supplementation increased crypt depth in the jejunum compared with the NC group (*P* < 0.05). The villus height-to-crypt depth ratio in the jejunum and ileum was significantly elevated by dietary *L. reuteri XY227* administration (Fig. 3C, *P* < 0.05).

### Gut microbiota in the ileum mucosa

**Fig. 4:**
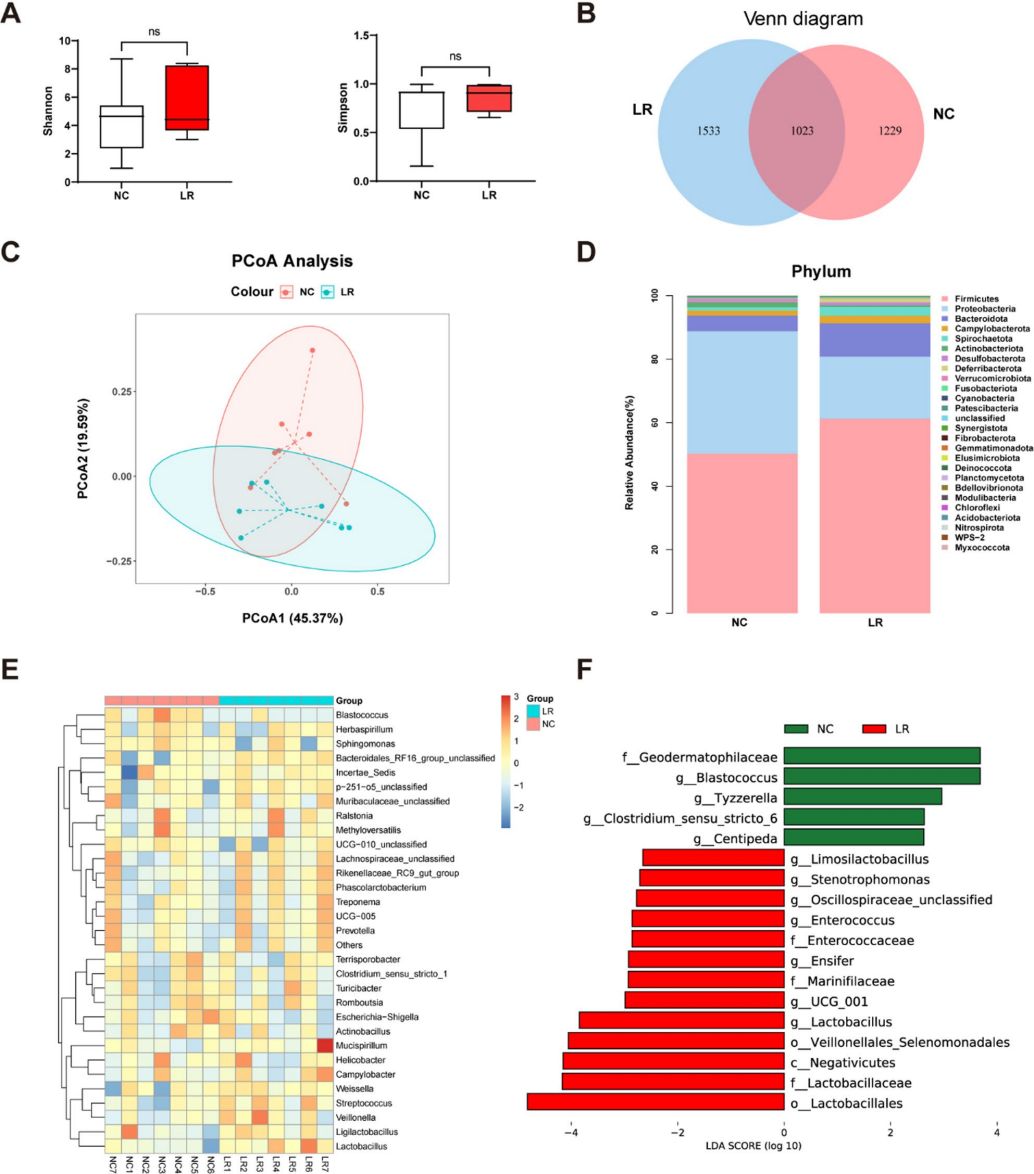
The microbial community in ileum mucosa (*n* = 7). (**A**) Alpha diversity (**B**)Veen diagram (**C**) PCoA of the ileum mucosa microbiome based on weighted the UniFrac distance metrics. (**D**) The phylum level of microbiota (**E**) The mean relative abundance of top 30 genera in the ileum mucosa. (**F**)The most differential taxa at genus level were exhibited by LEfSe analysis, LDA > 2.5. NC: basal diet, LR: basal diet supplemented with 0.4% *L. reuteri XY227* (1 × 10^11^ CFU/kg of basal diet)

We evaluated the impact of *L. reuteri XY227* supplementation on the microbial communities of the ileum mucosa. The Shannon index and Simpson index of mucosa microbiota were unaffected by *L. reuteri XY227* supplementation (Fig. 4A). Analysis of gut microbiota composition revealed 1023 shared operational taxonomic units (OTUs) between both groups. However, the LR group exhibited a higher total number of OTUs (1533) compared with the NC group (1229) (Fig. 4B). PCoA showed distinct clustering patterns between the NC and LR groups for ileal mucosa (Fig. 4C). At the phylum level, *Firmicutes*, *Proteobacteria*, and *Bacteroidota* were dominant in both groups, accounting for 90% of the relative abundance (Fig. 4D). LEfSe analysis identified differentially abundant bacterial genera between the NC and LR groups. At the family level, *L. reuteri XY227* increased the relative abundance of *Lactobacillaceae* and *Enterococcaceae* in the ileal mucosa of finishing pigs. At the genus level, *Lactobacillus*, *Enterococcus*, and *Limosilactobacillus* were significantly enriched in pigs treated with *L. reuteri XY227* (Fig. 4F).

### Concentrations of colonic SCFAs

**Fig. 5:**
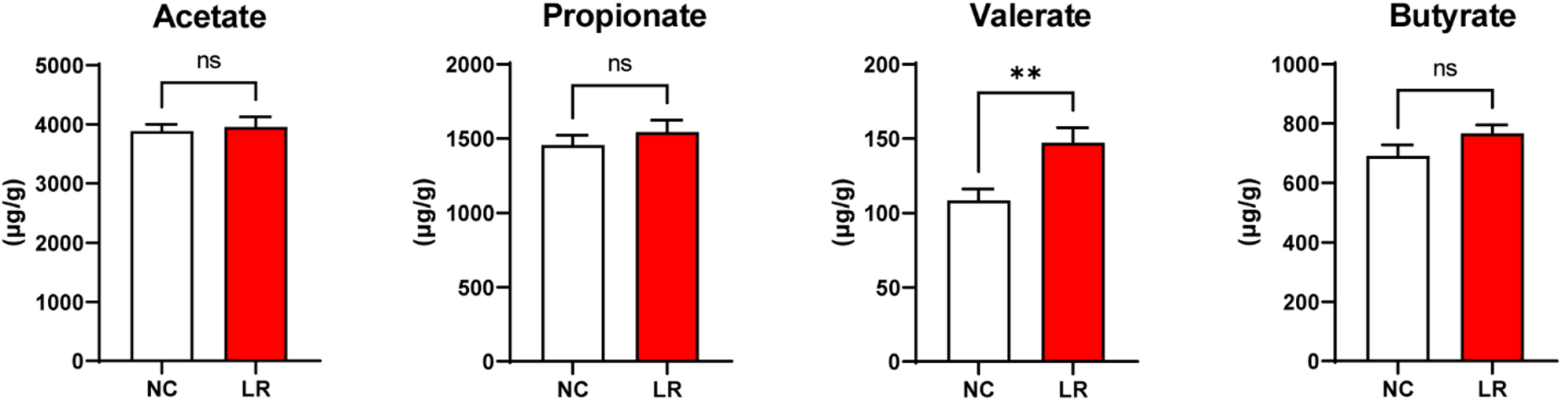
The concentration of SCFAs in colon of finishing pigs (*n* = 8). NC: basal diet, LR: basal diet supplemented with 0.4% *L. reuteri XY227* (1 × 10^11^ CFU/kg of basal diet). Data are presented as means ± SEM. *P < 0.05, **P < 0.01, ***P < 0.001

The concentrations of colonic acetate, butyrate, and propionate did not differ significantly between the LR and NC groups. However, supplementation with *L. reuteri XY227* significantly increased the concentration of colonic valerate compared with the NC group (Fig. 5, *P* < 0.05).

## Discussion

Compared to commercial DLY pigs, Ningxiang pigs exhibit superior meat quality and higher intramuscular fat content. In a previous study comparing the gut microbiota of seven finishing pig breeds have demonstrated that Chinese indigenous pigs possess significantly stronger gut microbial lipid metabolism capabilities than DLY pigs [13]. This further suggests that host fat synthesis may be mediated by gut microbial lipid metabolism activity.

The *L. reuteri* strains isolated from piglet feces exhibit probiotic properties, enhancing growth performance and alleviating diarrhea in piglets [14, 15]. Additionally, *Lactobacillus* strains isolated from the intestines of healthy elderly individuals show significant differences from those derived from fermented food, suggesting that gut probiotics undergo long-term adaptive changes in the human host. Another study showed that the functionalities of Bifidobacterial species in the gut microbiome can be influenced by the host diet [16]. Collectively, these findings support the concept of host species specificity in probiotics. Therefore, in this study, we aimed to determine whether supplementation with *L. reuteri XY227*, derived from Ningxiang pigs, exerted specific probiotic effects on gut microbiota homeostasis and metabolite changes, thereby improving lipid metabolism and growth performance in lean pigs. Our results demonstrated that dietary *L. reuteri XY227* supplementation significantly increased the FBW and ADG of finishing pigs. This improvement in growth performance may be attributed to the observed enhancement of intestinal morphology, as *L. reuteri* XY227 significantly increased villus height and the villus height-to-crypt depth ratio in both the jejunum and ileum, potentially facilitating improved feed digestion and nutrient absorption.

Lipid metabolism involves lipid synthesis and degradation [2]. Several genes, including lipoprotein lipase (*LPL)*, hormone-sensitive lipase *(HSL)*, carnitine palmitoyltransferase 1 *(CPT-1)*,* ACC*,* PPARγ*, and *SREBP1-C*, are key regulators in this process [17–19]. *PPARγ*, in particular, is a master regulator of adipogenesis, which influences lipid and glucose metabolism by promoting fatty acid uptake and lipid storage [20–22]. In our study, treatment with *L. reuteri XY227* reduced the mRNA expression of genes associated with lipogenesis, including *SREBP1-C*, *ACC*, and *PPARγ*, in the adipose tissue. SREBP-1c crucially regulates lipid homeostasis by initiating de novo lipogenesis and fatty acid metabolism in the liver and adipose tissue [23]. Activated *SREBP1-C* stimulates fatty acid biosynthesis by upregulating the expression of lipogenesis-related genes, such as *ACC* [24]. *ACC* catalyzes the conversion of acetyl-CoA into malonyl-CoA and facilitates the de novo synthesis of fatty acids, thereby functioning as a major regulator of cellular lipid metabolism [25]. In our study, *L. reuteri XY227* supplementation significantly downregulated the expression of *SREBP-1c*, *ACC*, and *PPARγ* in dorsal subcutaneous adipose tissue. This was accompanied by notable alterations in the fatty acid composition, specifically marked reductions in lauric acid (C12:0), eicosadienoic acid (C20:2), Oleic acid (C18:1n9c), and MUFA. Saturated fatty acids, particularly C12:0, are known to activate the Toll-like receptor 4 (TLR4) signaling pathway, thereby promoting pro-inflammatory responses in adipose tissue [26]. The observed reduction in C12:0 suggests a potential reduction of TLR4-mediated inflammatory signaling [27]. In addition, the decrease in C18:1n9c and MUFA levels indicates a reduction in lipid deposition [28]. Collectively, these findings suggest that the suppression of lipogenic gene expression by *L. reuteri* XY227 reshaped the fatty acid profile.

Several membrane or membrane-associated proteins, including those in the fatty acid transport protein family, fatty acid binding proteins, and *CD36*, are involved in the uptake and transport of cellular fatty acids [29]. *CD36* is crucial for the uptake of long-chain fatty acids, contributing to intracellular lipid accumulation [30]. Our findings confirmed that *L. reuteri XY227* enhanced the expression of *CD36* in subcutaneous adipose tissue and the jejunum. Fatty acids function as energy sources and critical signaling molecules, linking metabolism and immunity [31]. In accordance with the current results, a previous research showed that the fatty acid profiles of subcutaneous adipose tissues in finishing pigs is predominantly composed of C18:1n9c, C16:0, C18:2n6c, and C18:0, collectively constituting over 90% of total fatty acids [32]. Interestingly, despite the upregulation of *CD36* expression in both the intestine and adipose tissue—indicating enhanced fatty acid uptake capacity—the levels of C12:0, C20:2, C18:1n9c, and MUFA in adipose tissue were significantly reduced. Although fatty acid transport may be enhanced, the metabolic fate of these lipids may shift from accumulation to utilization. Our previous study has demonstrated that *L. reuteri* XY227 can increase intramuscular fat content in DLY pigs [10]. Taken together, these findings suggest that *L. reuteri* XY227 may promote both fatty acid transport and utilization.

Intestinal morphology is a crucial indicator of intestinal health, with crypt depth, villus height, and their ratio serving as key measures of the pig’s ability to absorb nutrients from feed [33]. Previous study has reported that *L. reuteri* promotes intestinal epithelial cell proliferation by activating the Wnt/β-catenin signaling pathway [34]. Furthermore, *L. reuteri* enhances the production of SCFAs and suppresses potential pathogens, thereby providing a critical energy source for enterocytes and reducing intestinal barrier damage [35, 36]. Together, these mechanisms contribute to improved villus development and gut health. Our results indicated that the jejunal and ileal villus height-to-crypt depth ratios significantly increased in the LR group, suggesting enhanced nutrient absorption, which may contribute to the increased daily weight gain with *L. reuteri XY227* supplementation.

Probiotics can affect the gut and extraintestinal organs by modulating microbial community structures and metabolite production [2]. Notably, 16 S rDNA sequencing analysis revealed significant alterations in the ileal mucosa microbiota of finishing pigs treated with *L. reuteri XY227*, highlighted by distinct biomarkers. Supplementation with *L. reuteri XY227* significantly increased the abundance of dominant community members, including *Lactobacillus*, *Enterococcus*, and *Limosilactobacillus*, with *Lactobacillus* emerging as the predominant genus. The abundance of *Lactobacillus* correlated significantly with increased valerate content in the colon. SCFAs are primary metabolites produced by bacterial fermentation of dietary fibers in the gut and crucially modulate host functions, including intestinal function, metabolic health maintenance, and immune system development [37, 38]. Notably, *Lactobacillus* and *Limosilactobacillus*, which were significantly enriched in the *L. reuteri* group, are known producers of short-chain fatty acids (SCFAs), including valeric acid. Valerate has been reported to serve not only as an energy substrate but also as a signaling molecule that modulates adipocyte differentiation and fatty acid metabolism through G-protein-coupled receptors 41 and 43 [39, 40]. Although we analyzed microbial alterations in the ileum, previous studies have reported that antibiotic infusion into the terminal ileum affected specific jejunal and colonic microbial populations and decreased SCFA concentrations in the colon [41]. This indicates that changes in the gut microbiota are not confined to a single intestinal segment and that microbial changes in the small intestine can influence downstream fermentation and SCFA profiles in the colon. Therefore, we observed an increase in colonic valerate levels in the LR group, reflecting alterations in the gut microbiota composition, which promoted the expression of genes related to lipid metabolism and fatty acid transport. Together, these findings suggest that *L. reuteri* XY227 modulates host lipid metabolism by reshaping the ileal microbiota, increasing valerate production, and improving intestinal health and nutrient absorption capacity.

## Conclusion

In conclusion, dietary supplementation with *L. reuteri XY227* improved growth performance, lipid metabolism and gut health in finishing pigs. Supplementation with *L. reuteri XY227* regulated the expression of key genes involved in lipid metabolism, fatty acid uptake, and transport in subcutaneous adipose tissue. In addition, *L. reuteri XY227* enhanced intestinal morphology and promoted the absorption of fatty acids by modulating intestinal structure and SCFA metabolites. These findings highlight the potential of *L. reuteri XY227* as a functional probiotic for improving lipid metabolism and intestinal health in finishing pigs.

## Data Availability

Sequence data is stored in the SRA database (PRJNA1063880).

## Abbreviations

DLY: Duroc × Landrace × Yorkshire
IBW: Initial body weight
FBW: Final body weight
ADG: Average daily gain
ADFI: Average daily feed intake
DE: Digestible energy
NDF: Neutral detergent fiber
ADF: Acid detergent fiber
GLU: Glucose
ALP: Alkaline phosphatase
ALT: Alanine aminotransferase
AST: Aspartate aminotransferase
TP: Total protein
BUN: Blood urea nitrogen
ALB: Albumin
GLB: Globulin
PUFA: Polyunsaturated fatty acids
SCFA: Short-chain fatty acid
LPL: Lipoprotein lipase
HSL: Hormone-sensitive lipase
CPT-1: Carnitine palmitoyltransferase 1
SREBP1-C: Sterol regulatory element binding protein-1c
PPARγ: Peroxisome proliferator-activated receptorγ
ACLY: ATP-citrate lyase
ACC: Acetyl CoA carboxylase
C/EBP-α: CCAAT enhancer binding proteinα
CD36: Cluster of differentiation 36
FABP4: Fatty acid binding protein 4
FATP1: Fatty acid transport protein 1
GLUT4: Glucose transporter type 4

## References

[1] Yan H, Diao H, Xiao Y, Li W, Yu B, He J, et al. Gut microbiota can transfer fiber characteristics and lipid metabolic profiles of skeletal muscle from pigs to germ-free mice. Sci Rep. 2016;6:31786. 10.1038/srep31786. 27545196 10.1038/srep31786 PMC4992887

[2] Schoeler M, Caesar R. Dietary lipids, gut microbiota and lipid metabolism. Rev Endocr Metab Disord. 2019;20(4):461–72. 10.1007/s11154-019-09512-0. 31707624 10.1007/s11154-019-09512-0 PMC6938793

[3] Fei N, Zhao L. An opportunistic pathogen isolated from the gut of an obese human causes obesity in germfree mice. Isme J. 2013;7(4):880–4. 10.1038/ismej.2012.153. 23235292 10.1038/ismej.2012.153 PMC3603399

[4] Qiao S, Liu C, Sun L, Wang T, Dai H, Wang K, et al. Gut parabacteroides Merdae protects against cardiovascular damage by enhancing branched-chain amino acid catabolism. Nat Metab. 2022;4(10):1271–86. 10.1038/s42255-022-00649-y. 36253620 10.1038/s42255-022-00649-y

[5] Chen C, Fang S, Wei H, He M, Fu H, Xiong X, et al. Prevotella Copri increases fat accumulation in pigs fed with formula diets. Microbiome. 2021;9(1):175. 10.1186/s40168-021-01110-0. 34419147 10.1186/s40168-021-01110-0 PMC8380364

[6] Foley MH, O’Flaherty S, Allen G, Rivera AJ, Stewart AK, Barrangou R, Theriot CM. Lactobacillus bile salt hydrolase substrate specificity governs bacterial fitness and host colonization. Proc Natl Acad Sci USA. 2021;118(6). 10.1073/pnas.2017709118. 10.1073/pnas.2017709118 PMC8017965 33526676

[7] Hou C, Zeng X, Yang F, Liu H, Qiao S. Study and use of the probiotic Lactobacillus reuteri in pigs: a review. J Anim Sci Biotechnol. 2015;6(1):14. 10.1186/s40104-015-0014-3. 25954504 10.1186/s40104-015-0014-3 PMC4423586

[8] Li S, Qi C, Zhu H, Yu R, Xie C, Peng Y, et al. Lactobacillus reuteri improves gut barrier function and affects diurnal variation of the gut microbiota in mice fed a high-fat diet. Food Funct. 2019;10(8):4705–15. 10.1039/c9fo00417c. 31304501 10.1039/c9fo00417c

[9] Zheng F, Wang Z, Stanton C, Ross RP, Zhao J, Zhang H, et al. Lactobacillus rhamnosus FJSYC4-1 and Lactobacillus reuteri FGSZY33L6 alleviate metabolic syndrome via gut microbiota regulation. Food Funct. 2021;12(9):3919–30. 10.1039/d0fo02879g. 33977963 10.1039/d0fo02879g

[10] Yang M, Xie Q, Wang J, Zha A, Chen J, Jiang Q, et al. Ningxiang pig-derived Lactobacillus reuteri modulates host intramuscular fat deposition via branched-chain amino acid metabolism. Microbiome. 2025;13(1):32. 10.1186/s40168-024-02013-6. 39891238 10.1186/s40168-024-02013-6 PMC11786426

[11] Yin Y, Gong S, Han M, Wang J, Shi H, Jiang X, et al. Leucine regulates lipid metabolism in adipose tissue through adipokine-mTOR-SIRT1 signaling pathway and bile acid-microbe axis in a finishing pig model. Anim Nutr. 2023. 10.1016/j.aninu.2023.10.005. 38357569 10.1016/j.aninu.2023.10.005 PMC10864217

[12] Yang M, Yin Y, Wang F, Zhang H, Ma X, Yin Y, et al. Supplementation with lycium barbarum polysaccharides reduce obesity in High-Fat Diet-Fed mice by modulation of gut microbiota. Front Microbiol. 2021;12:719967. 10.3389/fmicb.2021.719967. 34512598 10.3389/fmicb.2021.719967 PMC8427603

[13] Hu J, Chen J, Ma L, Hou Q, Zhang Y, Kong X, et al. Characterizing core microbiota and regulatory functions of the pig gut Microbiome. Isme J. 2024;18(1). 10.1093/ismejo/wrad037. 10.1093/ismejo/wrad037 PMC10873858 38366194

[14] Wang Z, Wang L, Chen Z, Ma X, Yang X, Zhang J, Jiang Z. In vitro evaluation of Swine-Derived Lactobacillus reuteri: probiotic properties and effects on intestinal Porcine epithelial cells challenged with enterotoxigenic Escherichia coli K88. J Microbiol Biotechnol. 2016;26(6):1018–25. 10.4014/jmb.1510.10089. 26907754 10.4014/jmb.1510.10089

[15] Collins SL, Stine JG, Bisanz JE, Okafor CD, Patterson AD. Bile acids and the gut microbiota: metabolic interactions and impacts on disease. Nat Rev Microbiol. 2023;21(4):236–47. 10.1038/s41579-022-00805-x. 36253479 10.1038/s41579-022-00805-x PMC12536349

[16] Yin P, Zhang C, Du T, Yi S, Yu L, Tian F, et al. Meta-analysis reveals different functional characteristics of human gut bifidobacteria associated with habitual diet. Food Res Int. 2023;170:112981. 10.1016/j.foodres.2023.112981. 37316017 10.1016/j.foodres.2023.112981

[17] Sun C, Li A, Wang H, Ma J, Hou J. Positive regulation of acetate in adipocyte differentiation and lipid deposition in obese mice. Nutrients. 2023;15(17). 10.3390/nu15173736. 10.3390/nu15173736 PMC10489952 37686768

[18] Lim SH, Lee HS, Han HK, Choi CI. Saikosaponin A and D inhibit adipogenesis via the AMPK and MAPK signaling pathways in 3T3-L1 adipocytes. Int J Mol Sci. 2021;22(21). 10.3390/ijms222111409. 10.3390/ijms222111409 PMC8583978 34768840

[19] Chu H, Du C, Yang Y, Feng X, Zhu L, Chen J, Yang F. MC-LR aggravates liver lipid metabolism disorders in obese mice fed a high-fat diet via PI3K/AKT/mTOR/SREBP1 signaling pathway. Toxins (Basel). 2022;14(12). 10.3390/toxins14120833. 10.3390/toxins14120833 PMC9784346 36548730

[20] Montaigne D, Butruille L, Staels B. PPAR control of metabolism and cardiovascular functions. Nat Rev Cardiol. 2021;18(12):809–23. 10.1038/s41569-021-00569-6. 34127848 10.1038/s41569-021-00569-6

[21] Kökény G, Calvier L, Hansmann G. PPARγ and TGFβ-major regulators of metabolism, Inflammation, and fibrosis in the lungs and kidneys. Int J Mol Sci. 2021;22(19). 10.3390/ijms221910431. 10.3390/ijms221910431 PMC8508998 34638771

[22] Mirza AZ, Althagafi II, Shamshad H. Role of PPAR receptor in different diseases and their ligands: physiological importance and clinical implications. Eur J Med Chem. 2019;166:502–13. 10.1016/j.ejmech.2019.01.067. 30739829 10.1016/j.ejmech.2019.01.067

[23] Lee G, Kim YY, Jang H, Han JS, Nahmgoong H, Park YJ, et al. SREBP1c-PARP1 axis tunes anti-senescence activity of adipocytes and ameliorates metabolic imbalance in obesity. Cell Metab. 2022;34(5):702–e185. 10.1016/j.cmet.2022.03.010. 35417665 10.1016/j.cmet.2022.03.010

[24] Sheng D, Zhao S, Gao L, Zheng H, Liu W, Hou J, et al. BabaoDan attenuates high-fat diet-induced non-alcoholic fatty liver disease via activation of AMPK signaling. Cell Biosci. 2019;9:77. 10.1186/s13578-019-0339-2. 31548878 10.1186/s13578-019-0339-2 PMC6751621

[25] Yeudall S, Upchurch CM, Seegren PV, Pavelec CM, Greulich J, Lemke MC et al. Macrophage acetyl-CoA carboxylase regulates acute inflammation through control of glucose and lipid metabolism. Sci Adv. 2022;8(47):eabq1984; 10.1126/sciadv.abq1984. 10.1126/sciadv.abq1984 PMC9683712 36417534

[26] Rogero MM, Calder PC. Obesity, Inflammation, Toll-Like receptor 4 and fatty acids. Nutrients. 2018;10(4). 10.3390/nu10040432. 10.3390/nu10040432 PMC5946217 29601492

[27] Lee JY, Ye J, Gao Z, Youn HS, Lee WH, Zhao L, et al. Reciprocal modulation of Toll-like receptor-4 signaling pathways involving MyD88 and phosphatidylinositol 3-kinase/AKT by saturated and polyunsaturated fatty acids. J Biol Chem. 2003;278(39):37041–51. 10.1074/jbc.M305213200. 12865424 10.1074/jbc.M305213200

[28] Zou Y, Wang YN, Ma H, He ZH, Tang Y, Guo L, et al. SCD1 promotes lipid mobilization in subcutaneous white adipose tissue. J Lipid Res. 2020;61(12):1589–604. 10.1194/jlr.RA120000869. 32978274 10.1194/jlr.RA120000869 PMC7707166

[29] Samovski D, Jacome-Sosa M, Abumrad NA. Fatty acid transport and signaling: mechanisms and physiological implications. Annu Rev Physiol. 2023;85:317–37. 10.1146/annurev-physiol-032122-030352. 36347219 10.1146/annurev-physiol-032122-030352 PMC13221695

[30] Shu H, Peng Y, Hang W, Nie J, Zhou N, Wang DW. The role of CD36 in cardiovascular disease. Cardiovasc Res. 2022;118(1):115–29. 10.1093/cvr/cvaa319. 33210138 10.1093/cvr/cvaa319 PMC8752351

[31] Kimura I, Ichimura A, Ohue-Kitano R, Igarashi M. Free fatty acid receptors in health and disease. Physiol Rev. 2020;100(1):171–210. 10.1152/physrev.00041.2018. 31487233 10.1152/physrev.00041.2018

[32] Liu Y, Peng Y, Chen C, Ren H, Zhu J, Deng Y, et al. Flavonoids from mulberry leaves inhibit fat production and improve fatty acid distribution in adipose tissue in finishing pigs. Anim Nutr. 2023. 10.1016/j.aninu.2023.11.003. 38357574 10.1016/j.aninu.2023.11.003 PMC10864206

[33] Tang X, Xiong K, Fang R, Li M. Weaning stress and intestinal health of piglets: A review. Front Immunol. 2022;13:1042778. 10.3389/fimmu.2022.1042778. 36505434 10.3389/fimmu.2022.1042778 PMC9730250

[34] Wu H, Xie S, Miao J, Li Y, Wang Z, Wang M, Yu Q. Lactobacillus reuteri maintains intestinal epithelial regeneration and repairs damaged intestinal mucosa. Gut Microbes. 2020;11(4):997–1014. 10.1080/19490976.2020.1734423. 32138622 10.1080/19490976.2020.1734423 PMC7524370

[35] Mukhopadhya I, Louis P. Gut microbiota-derived short-chain fatty acids and their role in human health and disease. Nat Rev Microbiol. 2025;23(10):635–51. 10.1038/s41579-025-01183-w. 40360779 10.1038/s41579-025-01183-w

[36] Lin C, Zheng Y, Lu J, Zhang H, Wang G, Chen W. Differential reinforcement of intestinal barrier function by various Lactobacillus reuteri strains in mice with DSS-induced acute colitis. Life Sci. 2023;314:121309. 10.1016/j.lfs.2022.121309. 36563843 10.1016/j.lfs.2022.121309

[37] Nicolas GR, Chang PV. Deciphering the chemical lexicon of Host-Gut microbiota interactions. Trends Pharmacol Sci. 2019;40(6):430–45. 10.1016/j.tips.2019.04.006. 31079848 10.1016/j.tips.2019.04.006 PMC6681900

[38] Martin-Gallausiaux C, Marinelli L, Blottière HM, Larraufie P, Lapaque N. SCFA: mechanisms and functional importance in the gut. Proc Nutr Soc. 2021;80(1):37–49. 10.1017/s0029665120006916. 32238208 10.1017/S0029665120006916

[39] Canfora EE, Jocken JW, Blaak EE. Short-chain fatty acids in control of body weight and insulin sensitivity. Nat Rev Endocrinol. 2015;11(10):577–91. 10.1038/nrendo.2015.128. 26260141 10.1038/nrendo.2015.128

[40] Li S, Duan Y, Luo S, Zhou F, Wu Q, Lu Z. Short-chain fatty acids and cancer. Trends Cancer. 2025;11(2):154–68. 10.1016/j.trecan.2024.11.003. 39638744 10.1016/j.trecan.2024.11.003

[41] Zhang C, Peng Y, Mu C, Zhu W. Ileum terminal antibiotic infusion affects jejunal and colonic specific microbial population and immune status in growing pigs. J Anim Sci Biotechnol. 2018;9:51. 10.1186/s40104-018-0265-x. 29988607 10.1186/s40104-018-0265-x PMC6027559

